# Plant biotransformation of T2 and HT2 toxin in cultured organs of *Triticum durum* Desf

**DOI:** 10.1038/s41598-019-50786-w

**Published:** 2019-10-04

**Authors:** Laura Righetti, Tania Körber, Enrico Rolli, Gianni Galaverna, Michele Suman, Renato Bruni, Chiara Dall’Asta

**Affiliations:** 1 Department of Food and Drug, University of Parma, Viale delle Scienze 17/A, I-43124 Parma, Italy; 2 Chair of Analytical Food Chemistry, Technical University of Munich, Max-von-Imhof-Forum 2, D-85354 Freising, Germany; 3 Hamburg School of Food Science, Institute of Food Chemistry, University of Hamburg, Martin-Luther-King-Platz 6, D-20146 Hamburg, Germany; 4 Department of Department of Chemistry, Life Sciences and Environmental Sustainability, University of Parma, Via G.P. Usberti 11/a, Parma, Italy; 5 Barilla G.R. F.lli SpA, Advanced Laboratory Research, via Mantova 166, Parma, Italy

**Keywords:** Secondary metabolism, Mass spectrometry

## Abstract

The present study aimed at elucidating the uptake and biotransformation of T2 and HT2 toxins in five cultivars of durum wheat, by means of cultured plant organs. An almost complete absorption of T2 toxin (up to 100 µg) was noticed after 7 days, along with the contemporaneous formation of HT2 in planta, whereas HT2 showed a slower uptake by uninfected plant organs. Untargeted MS-analysis allowed to identify a large spectrum of phase I and phase II metabolites, resulting in 26 T2 and 23 HT2 metabolites plus tentative isomers. A novel masked mycotoxin, 3-acetyl-HT2-glucoside, was reported for the first time in wheat. The *in vitro* approach confirmed its potential to both investigate the contribution of plant metabolism in the biosynthesis of masked mycotoxins and to foresee the development of biocatalytic tools to develop nature-like mixtures to be used as reference materials.

## Introduction

The gradual understanding of the working principles involved in multitrophic interactions is emphasizing the chemical interplay between plants and microorganisms. Since its discovery, the close relationship involving the soil microbiota and plant roots revealed the existence of large, unexpected chemical networks, leading to the “wood wide web” concept^1^. These networks can provide a two-way exchange for a variety of organic compounds translocated by a “transportome”, mostly localized in the outer-lateral plasma membrane of root epidermal cells. Its role concerns the release and (re)uptake of photosynthates exudated in excess, along with flavonoids, coumarins and brassinosteroids acting as a mutual biochemical feedback between a plant and its associated microflora^2–4^. These connections sustain symbiotic-like relationships related to the holobiont definition and involving microorganisms growing in the proximity of both the rhizosphere and the phyllosphere, i.e. in the space surrounding roots and on the surface of epigean organs^5^.

To cope with the evolutive pressure induced by exogenous organic substances, a complex enzymatic pool is active in most plants. Most xenobiotics may be in fact biotransformed by reductive phase I metabolism and conjugated by phase II metabolism through regio-selective and stereospecific reactions mediated by P450 cytochromes and other plant enzymes^6^. The resulting metabolites may then be deposited in the apoplast, bound to cell wall or segregated in the vacuole, often with the final aim of their expulsion during leaf or tegumental senescence^7,8^.

Mycotoxins are fungal metabolites with a preeminent role in plant infection and whose relevance for human health is paramount, given their combination of toxicity and widespread distribution in key foods and crops. These substances are directly inoculated in plant cells, but may be also biosynthesized in soil in minor amounts. Once entered in plants, mycotoxins may undergo the same biotrasformation pathways mentioned above, with the formation of a variety of compounds usually described as masked mycotoxins^9^. Although many studies have been presented over the las decade, covering mainly *Fusarium* mycotoxins i.e. deoxynivalenol^9^, zearalenone^10,11^, and T2 and HT-2 toxins^12,13^, the development of comprehensive screenings of masked mycotoxins in foods and the precise evaluation of *in vivo* bioactivity is made difficult by limitations, including the lack of readily available reference materials^14,15^. Nevertheless, international authorities including the European Food Safety Authority are trying to improve the related regulation and enforce stricter controls, increasing the number of masked mycotoxins analyzed and fostering an improved knowledge on toxicological effects.

To fill the gap in the availability of reference materials and given their natural proclivity to absorb and biotransform these substances, both plant cell, organ cultures or entire plants may be investigated as potential biofactories, allowing at the same time the collection of further knowledge regarding the chemical interplay between plants and their pathogens. Despite the availability of powerful metabolomics approaches, few protocols are available to evaluate the feasibility of such process. Recent investigations have highlighted the presence of a clear physiological response in crops as in the case of wheat externally treated with deoxynivalenol, thus leading to hypothesize that at least such compound may be absorbed by the epidermis of healthy plants^16^. Similarly, the absorption of zearalenone by isolated leaves and roots and by entire micropropagated wheat plantlets highlighted an intense uptake capability and an extensive organo-selective biotransformation that may represent a viable starting point for the biocatalytic production of modified zearalenone^10,17^. In some cases, such plant-based approach revealed the existence of masked mycotoxins not known before and allowed a clearer distinction between biotransformations mediated by the “green liver” of plants and those regulated by the fungal secondary metabolism^11,18^.

In this work, a model based wheat organ cultures was applied to elucidate the uptake and metabolic fate of T2 and HT2 in durum wheat combined with a targeted-untargeted metabolomics approach. Five wheat varieties namely Cysco, Iride, Kofa, Normanno and Svevo were chosen. Our driven hypothesis was based on the possible disclosure of cultured organ potential as a biocatalytic tool for the production of masked mycotoxins, as well as a replicable model for the investigation of the interplay between mycotoxins and wheat physiology. The goals included both a comprehensive description of biotransformation pathways of T2 and HT2 in healthy wheat plants, a screening of the physiological response of plant metabolism to their exposure and a preliminary evaluation of the possible biotechnological exploitation of the model.

## Results

### Uptake

**Figure 1:**
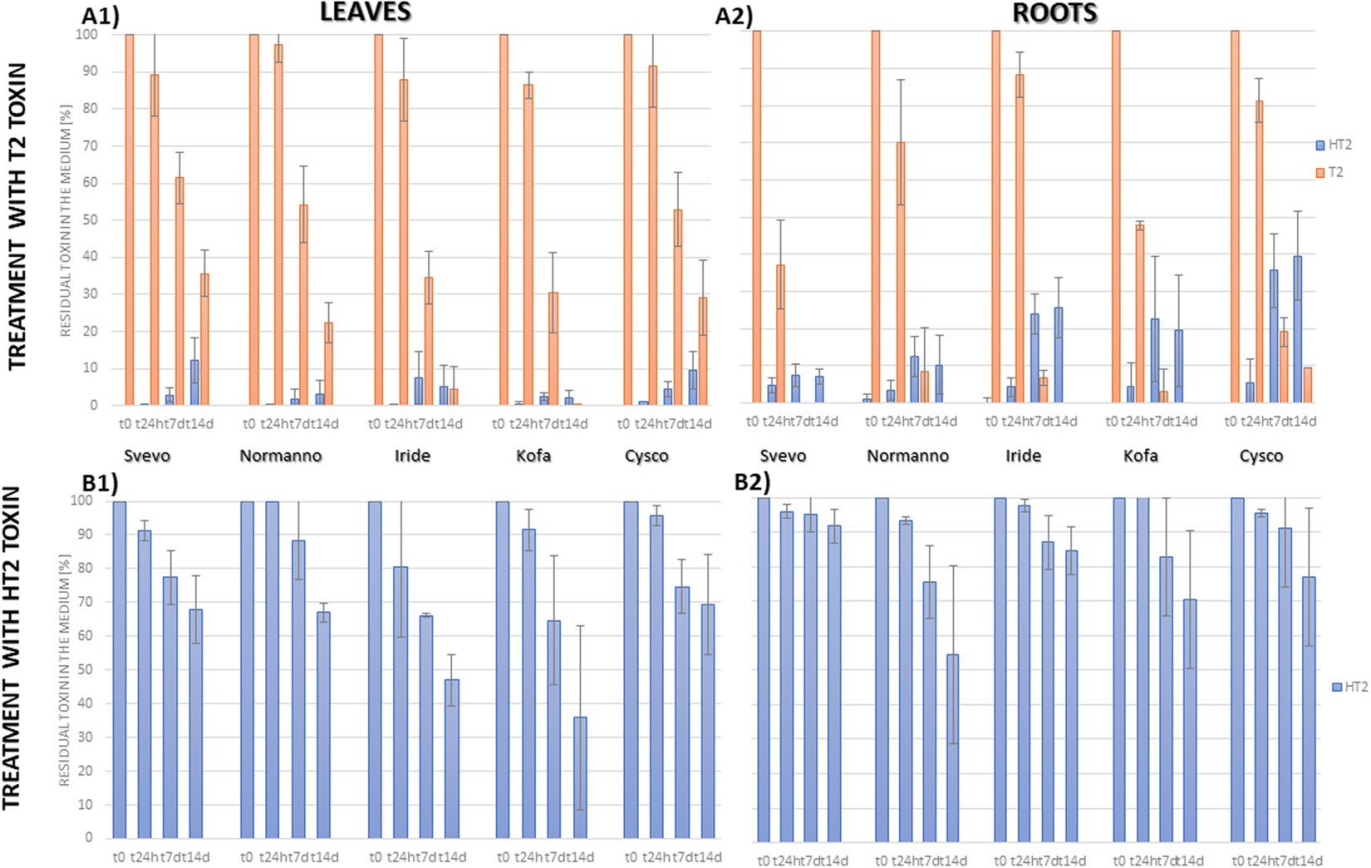
Residual T2 and HT2 (expressed as percentage, %, n = 4) found in leaves and roots media at t0, t24h, t7d, and t14d, upon treatment with T2 (plots A1 and A2, respectively) and HT2 (plots B1 and B2, respectively). Initial amount of toxin per treatment: 100 µg.

Growth media were monitored during the administration of T2 and HT2, to evaluate their apparent uptake. Both mycotoxins were not detected in the medium nor in roots and leaves blanks. In addition, no degradation of T2 and HT2 occurred due to chemical and physical agents (medium constituents and pH, temperature, light) during the whole experiment (14 days). In both roots and leaves, with the sole exception of cv. Svevo, T2 was completely removed after 14 days and in one case yet after 7 days (see Fig. 1, **plots A1** and **A2**). By comparison of the trendlines of the different experiments, T2 in the medium appeared to be quickly taken up by roots while a slower uptake was observed for the leaves, with the sole cv. Kofa showing a complete absorption after 14 days. On the contrary, the removal of HT2 from the medium was slower and less efficient in both organs (see Fig. 1, **plots B1** and **B2**). In general, the absorption was more efficient in Kofa and less in Svevo. The latter shown also visual symptoms of phytotoxicity (see Supplementary Information, Fig. 1S) after 14 days of HT2 treatment.

### Biotransformation

After 14 days of incubation with T2 or HT2, leaves and roots were analyzed by UHPLC-HRMS to evaluate their respective biotransformation and potential differences among cultivars. A total number of 26 and 23 metabolites plus tentative isomers were annotated for T2 and HT2 and listed in Tables 1 and 2, respectively.

**Table 1:** Metabolites of T2 annotated from roots and leaves analysis.

T2 metabolites	Formula	RT (min)	Detected *m/z* [M + NH4]^+^	Mass error (ppm)
15-acetyl-T2-tetraol-Glc^b^	C_23_H_34_O_12_	1.7	520.2403	2.8
15-acetyl-T2-tetraol-MalGlc^b^	C_26_H_36_O_15_	2.6	606.2404	1.9
dehydro-15-acetyl-T2-tetraol-Glc^c^	C_23_H_32_O_12_	3.2	518.2228	−0.7
Hydroxy-HT2-triGlc^c^	C_40_H_62_O_23_	3.7	944.3981	1.2
	C_40_H_62_O_23_	3.8	944.3941	−3
hydroxy-HT2-Mal-diGlc^b^	C_37_H_54_O_22_	4.4	868.3479	3.9
	C_37_H_54_O_22_	4.6	868.3445	0
	C_37_H_54_O_22_	4.6	868.3451	0.7
	C_37_H_54_O_22_	4.7	868.3463	2
	C_37_H_54_O_22_	4.9	868.347	2.9
	C_37_H_54_O_22_	5.1	868.3443	−0.2
hydroxy-HT2-diGlc^b^	C_34_H_52_O_19_	4.1	782.3469	2.8
	C_34_H_52_O_19_	4.2	782.3443	0.3
hydroxy-HT2-Glc^b^	C_28_H_42_O_14_	4.5	620.2921	1.2
hydroxy-HT2-MalGlc^b^	C_31_H_44_O_17_	5.1	706.2933	2.2
T2-triol-diGlc^c^	C_20_H_30_O_7_	5.6	724.3387	0.1
	C_20_H_30_O_7_	6.2	724.3391	0.6
hydroxy-HT2^c^	C_22_H_32_O_9_	5.8	458.2390	1.2
HT2-tri-Glc^b^	C_40_H_64_O_23_	6.1	928.4044	2.5
	C_40_H_64_O_23_	6.3	928.4045	2.7
T2-triol-Glc^b^	C_26_H_40_O_12_	6.3	562.2865	0.7
HT2-diGlc^b^	C_34_H_52_O_18_	6.7	766.3505	1.3
	C_34_H_52_O_18_	7.1	766.3484	−1
T2-triol^c^	C_20_H_30_O_7_	6.7	400.2340	2.6
dehydro-HT2-Glc^b^	C_28_H_40_O_13_	6.6	602.2801	−0.6
	C_28_H_40_O_13_	7.1	602.2830	3.8
HT2-Mal-diGlc^b^	C_37_H_54_O_21_	6.9	852.3513	2
	C_37_H_54_O_21_	7.1	852.3503	0.8
	C_37_H_54_O_21_	7.2	852.3478	−2.1
	C_37_H_54_O_21_	7.5	852.3500	0.5
	C_37_H_54_O_21_	8	852.3505	1.1
HT2-HexPent^b^	C_33_H_50_O_7_	7	736.3401	2
	C_33_H_50_O_7_	7.4	736.3378	−1.2
HT2-Glc^b^	C_28_H_42_O_13_	7.5	604.2967	0.5
	C_28_H_42_O_13_	7.6	604.2972	1.3
Hydroxy-HT2-diMal-diGlc^b^	C_40_H_56_O_24_	7.8	938.3477	−2.5
	C_40_H_56_O_24_	8.2	938.3474	−2.8
	C_40_H_56_O_24_	8.7	938.3468	−3.4
HT2^a^	C_22_H_32_O_8_	8.4	442.2452	3.8
HT2-MalGlc^b^	C_31_H_44_O_16_	8.1	690.2968	0.1
	C_31_H_44_O_16_	8.4	690.2952	−2.2
	C_31_H_44_O_16_	8.3	690.2961	−0.1
HT2-anhydro-HexGlc^b^	C_34_H_50_O_17_	8.6	748.3414	2.8
3-acetyl-HT2-Glc^b^	C_30_H_44_O_14_	8.8	646.3087	2.4
T2^a^	C_24_H_34_O_9_	10.2	484.2549	1.7
3-acetyl-T2^b^	C_26_H_36_O_10_	12.7	526.2626	−3.9
feruloyl-T2^b^	C_34_H_42_O_12_	14.4	660.3003	−1.6
	C_34_H_42_O_12_	14.3	660.3024	1.5

^a^Confirmation with standard by comparison of accurate mass, HRMS/MS and RT.

^b^Annotation with accurate mass, elemental formula and HRMS/MS spectra.

^c^Annotation with accurate mass and elemental formula.

**Table 2:** Metabolites of HT2 annotated from roots and leaves analysis.

HT2 metabolites	Formula	RT (min)	Detected *m/z* [M+NH4]^+^	Mass error (ppm)
15-acetyl-T2-tetraol-Glc^b^	C_23_H_34_O_12_	1.7	520.2403	2.8
15-acetyl-T2-tetraol-MalGlc^b^	C_26_H_36_O_15_	2.6	606.2404	1.9
dehydro-15-acetyl-T2-tetraol-Glc ^c^	C_23_H_32_O_12_	3.2	518.2228	−0.7
hydroxy-HT2-diGlc^b^	C_34_H_52_O_19_	4.1	782.3469	2.8
	C_34_H_52_O_19_	4.2	782.3443	0.3
hydroxy-HT2-Glc^b^	C_28_H_42_O_14_	4.5	620.2921	1.2
hydroxy-HT2-MalGlc^b^	C_31_H_44_O_17_	5.1	706.2933	2.2
hydroxy-HT2-Mal-diGlc^b^	C_37_H_54_O_22_	4.4	868.3479	3.9
	C_37_H_54_O_22_	4.6	868.3445	0
	C_37_H_54_O_22_	4.7	868.3463	2
	C_37_H_54_O_22_	4.9	868.3470	2.9
	C_37_H_54_O_22_	5.1	868.3443	−0.2
T2-triol-diGlc^c^	C_20_H_30_O_7_	5.6	724.3387	0.1
	C_20_H_30_O_7_	6.2	724.3391	0.6
hydroxy-HT2^c^	C_22_H_32_O_9_	5.8	458.2390	1.2
T2-triol-Glc^b^	C_26_H_40_O_12_	6.3	562.2865	0.7
T2-triol^c^	C_20_H_30_O_7_	6.7	400.2340	2.6
dehydro-HT2-Glc^b^	C_28_H_40_O_13_	6.6	602.2801	−0.6
	C_28_H_40_O_13_	7.1	602.2830	3.8
HT2-diGlc^b^	C_34_H_52_O_18_	6.7	766.3505	1.3
	C_34_H_52_O_18_	7.1	766.3484	−1
HT2-Mal-diGlc^c^	C_37_H_54_O_21_	6.9	852.3513	2
	C_37_H_54_O_21_	7	852.3503	0.8
	C_37_H_54_O_21_	7.2	852.3478	−2.1
	C_37_H_54_O_21_	7.5	852.3500	0.5
	C_37_H_54_O_21_	8	852.3505	1.1
HT2-HexPent^b^	C_33_H_50_O_7_	7	736.3401	2
	C_33_H_50_O_7_	7.4	736.3378	−1.2
HT2-Glc^b^	C_28_H_42_O_13_	7.5	604.2967	0.5
	C_28_H_42_O_13_	7.6	604.2972	1.3
HT2-MalGlc^b^	C_31_H_44_O_16_	8.1	690.2968	0.1
	C_31_H_44_O_16_	8.3	690.2961	−0.1
	C_31_H_44_O_16_	8.4	690.2952	−2.2
HT2^a^	C_22_H_32_O_8_	8.4	442.2452	3.8
HT2-anhydro-HexGlc^b^	C_34_H_50_O_17_	8.6	748.3414	2.8
3-acetyl-HT2-Glc^b^	C_30_H_44_O_14_	8.8	646.3087	2.4
3-acetyl-HT2^b^	C_24_H_34_O_9_	9.6	489.2546	−0.2
T2^a^	C_24_H_34_O_9_	9.8	484.2555	1.4
3-acetyl-T2^b^	C_26_H_36_O_10_	12.7	526.2626	−3.9

^a^Confirmation with standard by comparison of accurate mass, HRMS/MS and RT.

^b^Annotation with accurate mass, elemental formula and HRMS/MS spectra.

^c^Annotation with accurate mass and elemental formula.

The parent mycotoxins T2 or HT2 were found in both organs at lower amounts compared to other metabolites, confirming their intensive biotransformation.

Among the total annotated metabolites, 41 out of 49 were already reported in wheat^12^ and oat^13^, thus their identification was based on comparison of accurate mass and fragmentation pattern with those proposed by other authors. The remaining novel metabolites were tentatively identified by studying their HRMS/MS spectra. Only few metabolites (i.e Hydroxy-HT2-triGlc and T2-triol-diGlc) were tentatively annotated with accurate mass and elemental formula, since their intensities were too low to generate meaningful product ion spectra.

As a result, a large spectrum of hydroxylated and glycosylated forms of the mycotoxins, malonylglucosides and hexose-pentose conjugates, and acetyl and feruloyl metabolites were elucidated.

### Structure annotation of novel HT2 metabolite

**Figure 2:**
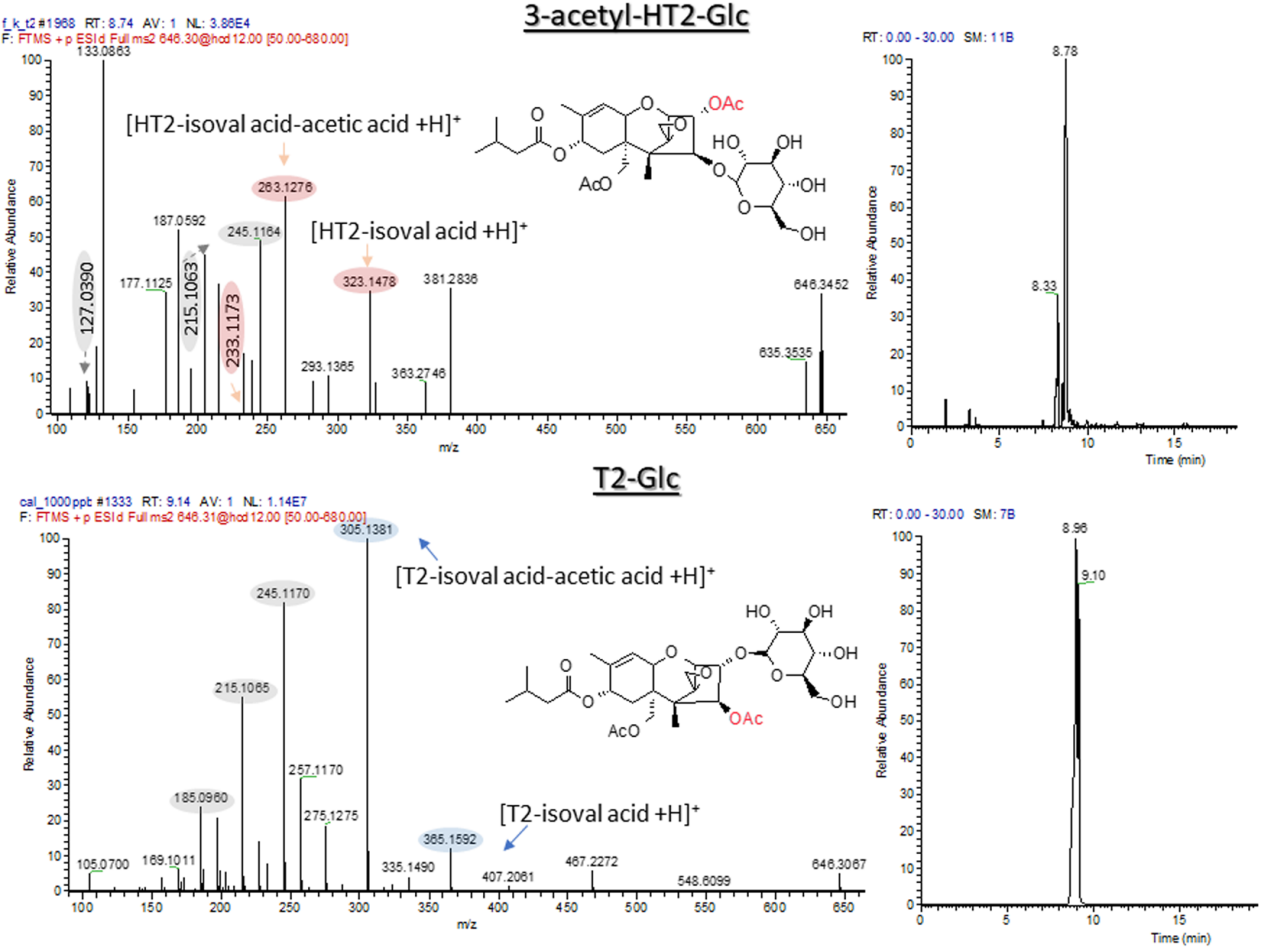
Comparison of EICs and LC-HRMS/MS spectrum of 3-Ac-HT2-Glc and T2-Glc isomers.

Among annotated metabolites, the formation of 3-acetyl-HT2-glucoside was reported for the first time in wheat. As reported in Fig. 2, the molecular ion *m/z* 640.3069, isobaric with T2-glucoside, was fragmented with a collision energy of 12 eV giving rise to typical T2 fragments (blue), HT2 characteristic fragments (pink) and T2/HT2 backbone common fragments (grey).

When looking for T2 glucoside in roots and leaves full scan mass spectrum, a peak fitting for the sum formula C_30_H_44_O_14_ (2.3 ppm mass deviation) was detected at the retention time of 8.8 min. By comparison of the retention time (8.96 and 9.10 min) and the HRMS/MS of T2-Glc of the standard and of the plant extract, differences were noticed.

Characteristic fragment ions of glucose (*m/z* 127.0390) and of the backbone namely *m/z* 185.0960, *m/z* 215.1057 and *m/z* 245.1164 were observed in both HRMS/MS metabolites, as shown in Fig. 2. In the of HRMS/MS spectrum of T2-Glc standard the fragment *m/z* 305.1369 was the most abundant and correspond to the loss of glucose, isovaleric acid and acetic acid from T2 [T2 − isoval acid − acetic acid + H] + . However, this fragment was not observed in organ samples, where the most abundant ion was *m/z* 263.1277 [HT2 − isoval acid − acetic acid + H]+. The absence of the fragment *m/z* 305.1369 can be explained by a more unstable bond of acetyl group at C-3, causing to a disintegrate immediately.

Owing to the obtained experimental mass of *m/z* 646.3087, the differences in the retention time and HRMS/MS between standard and plant material, it was postulated that the peak at 8.8 min could presumably be 3-acetyl-HT2-Glc. This assumption is also supported by the earlier elution of 3-acetyl-HT2-Glc (8.8 min) compared to its aglycone 3-acetyl-HT2 (9.6 min).

As a result, T2-Glc was not detected as a T2 plant metabolite, in agreement with Nathanail *et al*.^12^, while its was found in barley^19^.

### Organ-related metabolites formation

**Figure 3:**
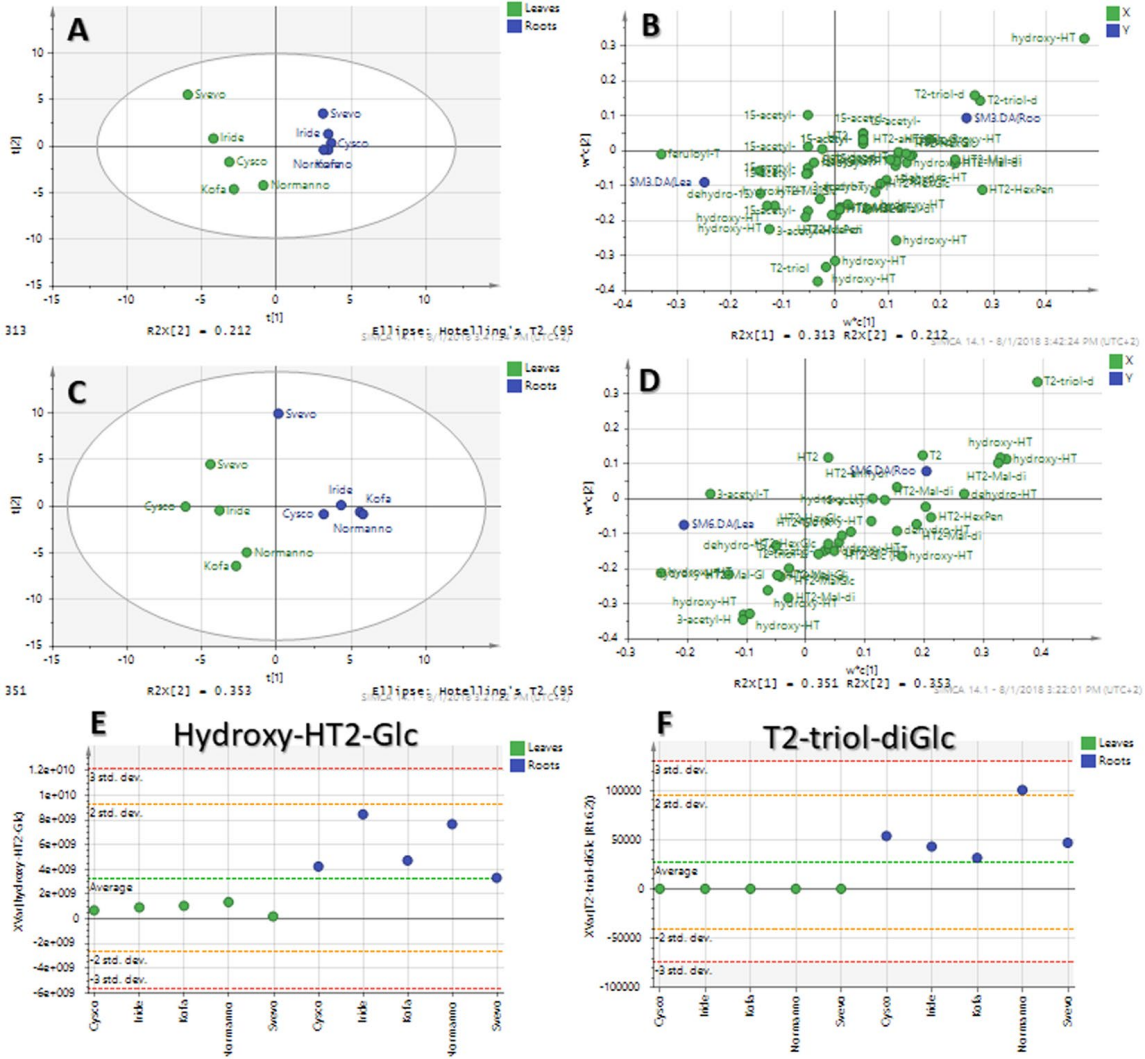
PLS-DA score scatter plots (panel A and B) and loading plots (panel C and D) for T2 (R2X = 0.597, R2Y = 0.995, Q2 = 0.917) and HT2 (R2X = 0.784, R2Y = 0.987, Q2 = 0.777). Trend plots (panel E and F) of the most significant metabolites of T2 (hydroxy-HT2-Glc) and HT2 (T2-triol-diGlc) experiments.

To assess the detoxification ability of the two plant organs, a PCA was built using the peak area of the identified metabolites. Samples were clustered according to the organ (root or leaf) suggesting that the metabolism of T2 and HT2 was influenced by the enzymatic pool of the two organs (see Supplementary Information, Fig. 2S). Then, PLS-DA and its loading plot were constructed to highlight those compounds able to discriminate between root and leaves (Fig. 3).

PLS-DA analysis showed a good clusterization among leaves and roots, confirming differences in the organ-related metabolism. When cultivars are observed, it can be noticed that leaves samples are more spread than roots samples, probably on account of a larger metabolite biodiversity. In addition, Svevo cv. showed a different behavior suggested by its distance from the cluster, confirming also its lower uptake from the media and its visual symptoms. The remaining four cultivars were tight clustered both in the T2 and HT2 models (Fig. 3, **panels A**,**C**, respectively).

In particular, hydroxy-HT2-Glc and T2-triol-diGlc were found out to be significantly more abundant in roots than in leaves as shown by the trend plots (Fig. 3, **panels E and F**, respectively). Hydroxy-HT2-Glc was the most discriminant marker for T2 experiment as suggested also by its position in the PLS-DA loading plot, while T2-triol-diGlc was the most significant metabolites able to discriminate HT2 roots and leaves. A further explanation may be related to the different nature of these organs and their tissues, with the cortical cylinder of roots operating both for water and solute uptake purposes and as an active filter for unwanted substances. T2 and HT2 absorbed by the symplastic route may in fact encounter enzymatic pools whose role is to prevent their transfer to the xylematic transport, by transforming them in substances more easily segregated in vacuoles or in the apoplast of parenchimatous root cells^20^.

## Discussion

The use of wheat organ cultures and micropropagated plants has already demonstrated its potential for the investigation of zearalenone biotransformation^11,17^. Besides the identification of a wide range of metabolites and the annotation of novel compounds, the use of *in vitro* models allows the detection of cultivar-related and organ-related differences in a relatively short time limiting the constraints related to biological variability. Although the information obtained cannot be directly translated into risk assessment, the generated knowledge is of great support in the understanding of biological pathways.

Considering the uptake of T-2 and HT-2 toxins, a strong difference in the residual amount of administered toxin was found in the medium, when treatments with T2 and HT2 are compared in both roots and leaves. It is known that plant uptake is strongly dependent on the physicochemical characteristics of xenobiotics, including Henry’s Law constant, water solubility, and octanol−water partition coefficient^21,22^. In particular, for neutral chemicals, the uptake from soil medium is usually driven by hydrophobicity (usually expressed as logPow), as the degree of uptake appears to be proportional to the octanol−water partition coefficient. Briggs *et al*.^21^ proposed that plant uptake of neutral chemicals can be represented by a Gaussian distribution where maximum translocation of chemicals can be seen at a logPow ∼1.78.

Accordingly, the different uptake of the two mycotoxins might be ascribable to their n-octanol-water partitioning coefficients (logPow of 2.10 and 1.57 for T2 and HT2, respectively), which suggest an easier passive absorption of T2 through the lipophilic cuticular layer on leaf epidermis and waxy surface of roots. In addition to the compound-related lipophilic/hydrophilic balance, differences in uptake observed between wheat cultivars can be explained by factors such as degree of root growth, transpiration rates, and the size and shape of the leaf material, as already reported by other studies^23,24^. These characteristics, although not specifically addressed within this work, can significantly vary between cultivars and environmental conditions.

Once absorbed from the media, T2 and HT2 underwent biotransformation at large extent. The parent mycotoxin T2 or HT2 were present in all tissues. The amount of accumulated T2, measured as relative abundance of the signal, is variable but represent small percentage that varies between 1% (roots) and 6% (leaves), while for HT2 7% of non-metabolized mycotoxins was found both in roots and leaves.

In particular, T2 toxin seems to be quickly deacetylated to HT2, being the latter partially excreted in the medium and partially further metabolized. In our study, the T2-to-HT2 conversion progressed rapidly, since already after 24 h HT2 was excreted back to the medium.

On the contrast, the potential reverse acetylation of HT2 was only slightly observed in HT2-treated organs, where T2 metabolites have been observed in the analysed samples. This was in agreement with previous studies by Nathanail *et al*.^12^, who observed the HT2-to-T2 conversion in plants only at a slow rate.

As a general remark, phase II conjugates represented the majority of biotransformation products found in organs. The same pattern of phase II metabolites was observed in T2- as well as in HT2-treated organs, again suggesting that T2 is quickly deacetylated to HT2. Indeed, conjugation of HT2 represents the major biotransformation reaction for both T2 and HT2 with percentages in terms of relative abundance ranging between 77% in HT2 treated roots and 85% in T2 treated roots.

Besides well-known phase II conjugation pathways, among them glycosylation also followed by malonylation, and sulfation, less common conjugates have been elucidated, such as acetyl and feruloyl metabolites. Our results are in accordance with the previous finding of Nathanail *et al*.^12^ that described that the majority of the HT2 metabolites in wheat were derived from HT2-Glc by additional conjugations. Malonylation seemed to be a preferential route of conjugation, as already observed for other mycotoxins such as zearelone^25^. Other major metabolites are HT2-HexGlc, HT2-HexPent, HT2-MalGlc, HT2-Mal-di-Glc, some of them already described in barley and oats^12,19^.

As already discussed, one of the major advantage of using *in vitro* models for studying the biotransformation of mycotoxins in plants is the easiness to investigate organ-related metabolic pathways, also in relation to the decrease in toxic activity of the target compounds. Furthermore, its high degree of standardization may be exploited to produce precise mixtures of T2 and HT2 plant metabolites to be used as reference materials for both toxicological and analytical purposes.

**Figure 4:**
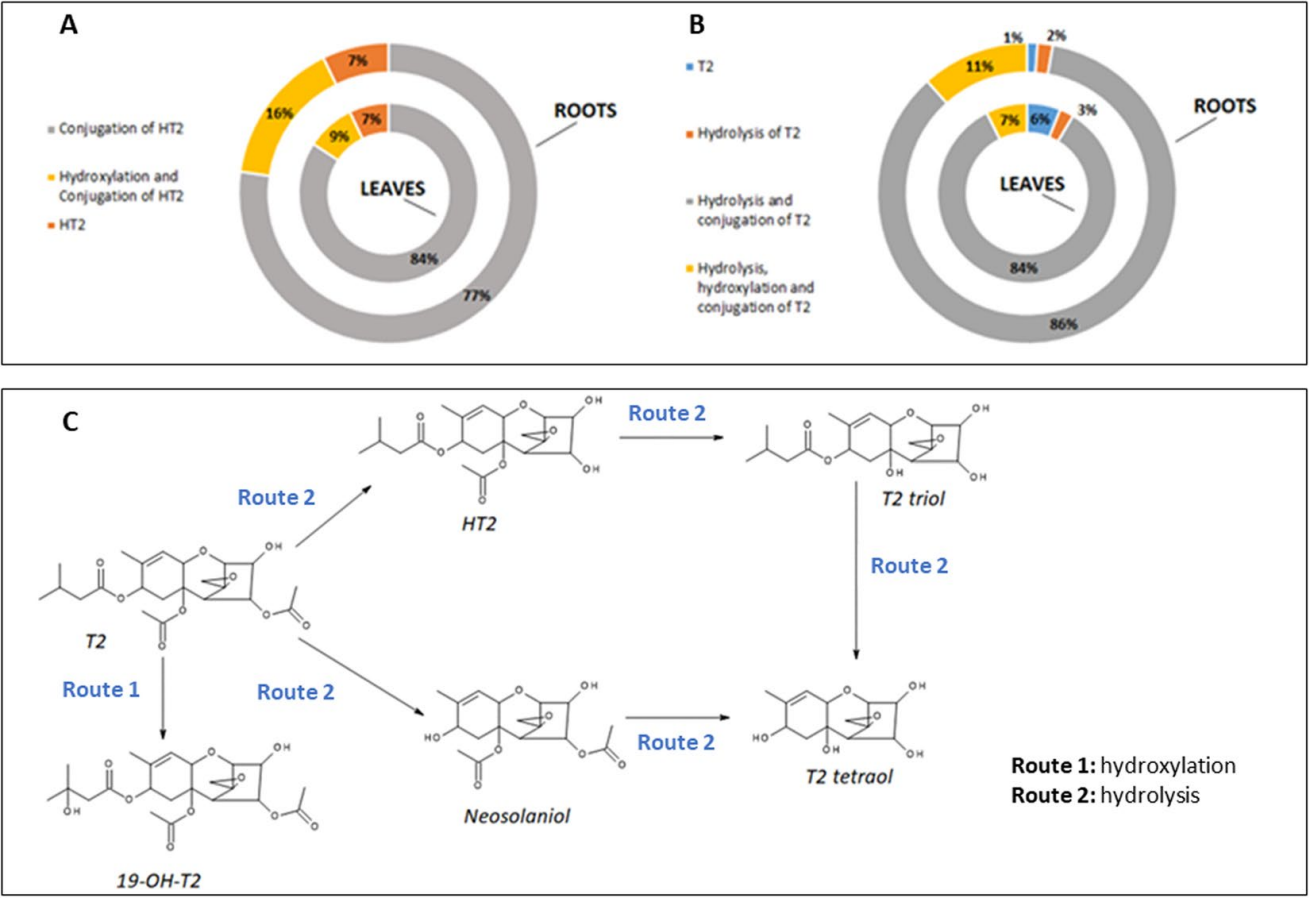
Distribution of phase I and phase II metabolites found in HT-2 and T2-treated roots and leaves (panel A and B, respectively). Main routes of formation of T2 phase I metabolites (panel C).

According to the recent EFSA opinion^26^, the biotransformation pathway of T2 and HT2 can be described by hydrolysis and hydroxylation events (phase I), followed by conjugation (phase II). In particular, the predominant metabolic pathway is the hydrolytic cleavage of one or more of the three ester groups of T2, as shown in Fig. 4, panel C. This is clearly mirrored in the wide spectrum of phase I and phase II metabolites accumulated in both roots and leaves and ascribable to a relevant hydrolytic activity. From our data, HT2-treated roots and leaves showed the accumulation of HT2-conjugates and, in minor proportion, of hydroxylated conjugates (Fig. 4, **panel A**). The quick biotransformation of HT2 suggests therefore that the compound likely undergoes direct phase II biotransformation in both organs. On the contrary, T2-treated roots and leaves strongly showed the activation of phase I pathways, leading to the quick hydrolysis of T2 to HT2, possibly followed by further hydroxylation, prior to conjugation (Fig. 4, **panel B**). In both HT2- and T2-treated samples, further hydrolysis to T2-triol and tetraol have been noticed as well.

Although data on the T2 and HT2 adverse effects on plants have received only limited attention^27^, it should be noticed that T2 and its modified forms are responsible for ribotoxicity, which seemed to be reduced by hydrolysis events (i.e. T2 > HT2 ≈ NEO > T2 treol > T2 tetraol)^28^. According to structure-activity relationship observations, the 12,13-epoxide group and the C9-C10 double bond are indeed crucial for exerting the ribotoxic effect, but the biological activity is modulated by the substituent group at C3, C4, and C15, as well. Concerning the hydroxyl group at C3, any conjugation leads to a decrease in the ability to bind the 60S ribosomal subunit, due to the hindrance related to a bulky substituent. Accordingly, acetylation at the C3 position of T2 may result in a loss of potency compared to the parent compound. On the contrary, the removal of the acetyl group at C4 and at C15 position, seems to strongly reduce the toxicity^29^. Normally, the activity of T2 toxin decreases when the substituent at C-8 position changes from an isovaleryloxy group to a hydroxyl group, while hydroxylation occurring at C3’ or C4’ does not affect the toxicity^29^. Therefore, organs treated with T2 toxin quickly activate both phase I and phase detoxification pathways, to effectively decrease the ribotoxicity of the compound and allow its compartmentalization in a more polar form. On the other side, when HT2 toxin is administered, conjugation may immediately take place with a reduced involvement of the phase I machinery. When the biotransformation pattern is compared between the tested cultivars, it can be noticed that Svevo cv. showed a slightly different behavior, as suggested by its distance from the PLS-DA cluster (Fig. 3), and by its lower mycotoxin uptake from the media (Fig. 1). This different behavior can be reflected in the observed visual symptoms as well. In particular, an activation of a back-acetylation route was observed only in Svevo cv. HT2-treated organs, leading to the formation of T2 and 3-acetyl-T2 in both roots and leaves. Similarly, T2-treated Svevo cv organs were characterized by a higher amount of feruoyl-T2 and lower accumulation of hydrolysed conjugates. Taken all together, these data suggest a lower activation of the detoxification pathways in Svevo cv. compared to other cultivars, in agreement with a more intensive phytotoxicity reported by visual observation of organs. Concerning other varieties considered within this work, few differences were noticed in terms of annotated metabolites. However, as general remark, a careful analysis of the PLS-DA data showed that leaf samples are more heterogeneous than root samples (see Fig. 3), thus indicating a larger biodiversity in the biotransformation pattern occurring in treated leaves than in roots. This is in agreement with other studies reporting on the differential activation of the defense machinery in roots and leaves of Graminaceae species as a response to abiotic and biotic stressors, resulting in a different expression of enzymatic pools^30,31^.

In conclusion, our study demonstrated that micropropagated organs are an efficient model for understanding the biotransformation of mycotoxins in plants, and for better investigate organ-related pathways. This model could be easily exploited in future works for the elucidation of defense mechanisms using a multi-omics approach based on integrated transcriptomics, proteomics and metabolomics studies. Comparative studies between different cultivars may support a deeper understanding of the molecular mechanisms underpinning inter- and intra-species resistance phenomena, and provide novel knowledge in support of breeding programs. Following its application to zearalenone metabolism in planta, the *in vitro* approach confirmed its feasibility to investigate the role of plants in the biosynthesis of masked mycotoxins and its potential as a biocatalytic tools to develop nature-like mixtures to be used as reference materials.

## Methods

### Chemicals and reagents

Analytical standards of T2 (100 µg mL^−1^ in acetonitrile) and HT2 (solution in acetonitrile 10 µg mL^−1^) were obtained from Sigma-Aldrich (Taufkirchen, Germany). T2 toxin glucosides were kindly provided by Dr. Susan P. McCormick (National Center for Agricultural Utilization Research, U.S. Department of Agriculture, Peoria, United States).

HPLC-grade methanol, acetonitrile and acetic acid, as well as dimethylsulfoxide (DMSO) were purchased from Sigma-Aldrich (Taufkirchen, Germany); bidistilled water was obtained using a Milli-Q System (Millipore, Bedford, MA, USA). MS-grade formic acid from Fisher Chemical (Thermo Fisher Scientific Inc., San Jose, CA, USA) and ammonium acetate (Fluka, Chemika-Biochemika, Basil, Switzerland) were also used.

### Plant material

Five commercial durum wheat (*Triticum durum Desf*.) varieties, namely Kofa, Svevo, Normanno, Iride and Cysco were selected among cultivars commonly used for pasta production. Characteristics are reported in Supplementary Information (Table 1S; data from Global Tetraploid Wheat Collection).

Kofa is a Desert Durum® variety developed by Western Plant Breeders (now West-Bred) that has excellent pasta quality with optimal semolina and pasta color, high protein content, and strong gluten^32^. Svevo, Normanno, Iride, and Cysco varieties are largely used in Italy and Southern Europe for superior pasta quality^33^. In particular, Svevo cv. and Normanno cv. are exclusively used in pasta industry.

Cultures and micropropagation of wheat were carried out as previously described^10^. All the experiments were carried out in triplicate and repeated three times.

For root induction, shoots were cultured on agarized MS medium hormone free. After 4 weeks, roots were excised and inoculated in liquid MS medium (50 ml) supplemented with 1 µM IBA in glass conical flasks (150 ml); cultures were kept in the dark under continuous agitation at 100 rpm in an orbital shaker and maintained in climatic chamber for 4 wk. To improve root growth, 1 µM IBA, as auxin, was added in the roots culture.

Leaves (3–5 cm in length) were excised from 3 weeks old plants and placed in 50 ml test tube containing few milliliters of solid MS medium. The leaf base was immersed into the medium, then the tubes were filled with liquid medium added with 10 µM N6-benzyladenine (BAP), sealed and incubated in climatic chamber for two weeks. Cytokinin BAP at 10 µM was present in the leaf culture medium to prevent tissue senescence.

### Sample preparation, T2 and HT2 administration, sampling and controls

T2 and HT2 were independently dissolved in an adequate amount of DMSO so that the final concentration of the solvent in culture medium did not exceed the one considered toxic (0.2%) with mycotoxin being at the final concentration of 100 µg/L.

Leaves and roots experiments were performed following the protocol previously described^10^. Liquid medium without mycotoxin was used in all experiments as a control.

To monitor the evolution of its absorption, T2 and HT2 presence in liquid media was quantified four times in both leaves and roots cultures and in flask containing solely liquid medium at the following intervals: t = 0, t = 1d, t = 7d and t = 14d. At the end of the experiment neither leaves nor roots cultures exposed to 100 µg/l T2 and HT2 showed any visible degradation. Plant material was harvested during day time and was immediately quenched in liquid nitrogen to deter any metabolic activity, then stored at −80 °C until further analysis.

Roots and leaves samples were homogenized and extracted with solvent mixture of acetonitrile/water/formic acid (79:20:1, v/v) and then subjected to LC-MS analysis^10^.

### Targeted UHPLC-MS/MS analysis of mycotoxins

UHPLC Dionex Ultimate 3000 separation system coupled to a triple quadrupole mass spectrometer (TSQ Vantage; Thermo Fisher Scientific Inc., San Jose, CA, USA) equipped with an electrospray source (ESI) was employed. For the chromatographic separation, a reversed-phase C18 Kinetex EVO column (Phenomenex, Torrance, CA, USA) with 2.10 × 100 mm and a particle size of 1.7 µm heated to 40 °C was used. 3 μl of sample extract was injected into the system; the flow rate was 0.4 ml/min. Gradient elution was performed by using 1 mM ammonium acetate in water (eluent A) and methanol (eluent B) both acidified with 0.5% acetic acid. Initial conditions were set at 10% B followed by a linear change to 40% B in 4 min and to 90% B in 16 min. Column was then washed for 2 min with 90% B followed by a reconditioning step for 3 min using initial composition of mobile phases. The total run time was 25 min. MS parameters: the ESI source was operated in positive ionization mode (ESI+); spray voltage 3,000 V, capillary temperature at 270 °C, vaporizer temperature was kept at 200 °C, sheath gas flow was set at 50 units and the auxiliary gas flow at 5 units. Detection was performed using multiple reaction monitoring (MRM) mode. The following optimized transitions were used for the quantification: T2 m/z 484 → 185 (CE = 22 eV); HT2 m/z 442 → 263 (CE = 11 eV); T2-Glc m/z 646 → 305 (CE = 15 eV). Quantification of target analytes in the medium samples was performed by using matrix matched calibration standard prepared by diluting blank medium with water/methanol (80:20, v/v) (calibration range 1–500 ng mL-1). Quantification was performed employing Thermo Xcalibur 2.2.SP1 QuanBrowser software.

### Untargeted HPLC-HRMS analysis

Roots and leaves extracts were subjected to HRMS in order to investigate the formation of T2 and HT2 biotransformation products, for which analytical standards were not available. Chromatographic conditions were the same used for the targeted analysis. LC-HRMS full scan spectra were recorded using Q-ExactiveTM high resolution mass spectrometer (Thermo Scientific, Bremen, Germany) equipped with electrospray ionization (ESI). The Q-Exactive mass analyzer was operated in the full MS/data dependent MS/MS mode (full MS–dd-MS/MS) in positive ionization mode. The following parameters were set: sheath and auxiliary gas flow rates 40 and 10 arbitrary units, respectively; spray voltage 3.5 kV; heater temperature 250 °C; capillary temperature 300 °C. Following parameters were used in full MS mode: resolution 70,000 FWHM (defined for *m/z* 200), scan range 400 ¬ 900 *m/z*, automatic gain control (AGC) target 3e6, maximum inject time (IT) 200 ms. Parameters for dd-MS/MS mode: resolution 17,500 FWHM (defined for m/z 200) AGC target 2e5, maximum IT 50 ms, normalized collision energy (NCE) 35% with ±25% step was used.

### Putative identification of T2 and HT2 metabolites

The full identification by comparison with of commercial standards has been performed only for T2 and HT2. Raw data files (roots and leaves in full scan-ddMS2 mode with and without an inclusion list) were imported into Compound Discoverer™ software (v. 1.0; Thermo Scientific, Fremont, CA, USA) to identify biotransformation metabolites. The software detects chromatographic peaks and the mass of the corresponding compound is compared with a list of generated theoretical metabolites (processing settings are summarized in the Supplementary Information, Table 2S, Figs 3S and 4S). Potential metabolites were described by exact mass, HRMS/MS fragmentation, isotopic pattern and retention time with respect to the parent compound (as determined from *in silico* predictions). Potential metabolites were further compared with literature reports^12,13,19^. Only in few cases, fragmentation spectra could not be collected, due to parent ion abundance below the threshold. In this case, a tentative annotation based on accurate mass and elemental formula was performed, as already proposed by other authors^12,13,19^. Potential and tentative metabolites are listed in Tables 1 and 2.

### Statistical analysis

Statistical analyses were performed using SIMCA software (v. 13.0, 2011, Umetrics, Umea, Sweden; www.umetrics.com). Metabolite intensities in roots and leaves were pre-processed using the pareto scaling and logarithmic transformation then unsupervised principal components analysis (PCA) models were built.

## Supplementary information

Supplementary Information

## Data Availability

https://data.mendeley.com/datasets/97hn6jphn4/draft?a=482014f0-05a2-42a0-9f0a-240d28f55168.
