# Supplementary material for: Plant biotransformation of T2 and HT2 toxin in cultured organs of Triticum durum Desf
Source: Sci Rep. 2019 Oct 4;9:14320. doi: 10.1038/s41598-019-50786-w (PMC6778183; doi:10.1038/s41598-019-50786-w)
Supplement: Supplementary file 1 — Supplementary Information [file 41598_2019_50786_MOESM1_ESM.docx]

**Supplementary Information for:**

**Plant biotransformation of T2 and HT2 toxin in cultured organs of *Triticum durum* Desf.**

Laura Righetti^1^, Tania Körber^2,3^, Enrico Rolli^4^, Gianni Galaverna^1^, Michele Suman^5^, Renato Bruni^1^, Chiara Dall’Asta^1*^

^1^Department of Food and Drug, University of Parma, Viale delle Scienze 17/A, I-43124 Parma, Italy

^2^Chair of Analytical Food Chemistry, Technical University of Munich, Max-von-Imhof-Forum 2, D-85354 Freising, Germany

^3^Hamburg School of Food Science, Institute of Food Chemistry, University of Hamburg, Martin-Luther-King-Platz 6, D-20146 Hamburg

^4^Department of Department of Chemistry, Life Sciences and Environmental Sustainability, University of Parma, Via G.P. Usberti 11/a, Parma, Italy

^5^Barilla G.R. F.lli SpA, Advanced Laboratory Research, via Mantova 166, Parma, Italy

*** Corresponding author:**Prof. Chiara Dall’Asta; [chiara.dallasta @unipr.it](mailto:laura.righetti@unipr.it)

**
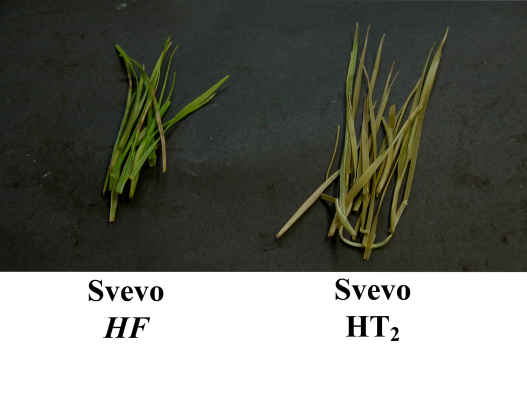

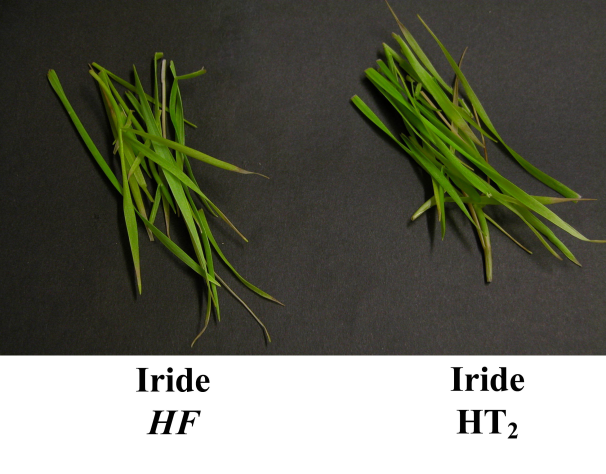
**

**Control**

**Control**

**Figure S1.** Visual symptoms of leaves treated with HT2 in Iride and Svevo varieties.

**
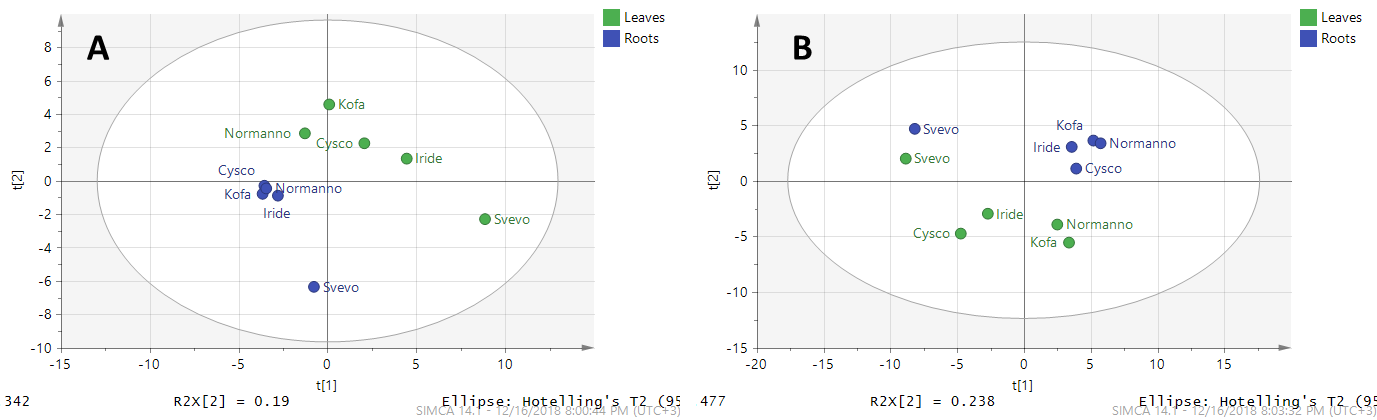
**

**Figure S2.** Principal Component Analysis (PCA) built using the peak area of the identified metabolites in both roots and leaves organs.

**Table S1:** Characteristics of durum wheat varieties used for this study

| **Durum wheat variety** | **Origin** | **Company** | **Heading** | **FHB** | **Registration** |
| --- | --- | --- | --- | --- | --- |
| Kofa | Desert, North America | West Bred | Late | Resistant | 1994 |
| Normanno | Northern Italy | Syngenta | Middle | Sensitive | 2002 |
| Svevo | Southern Italy | Syngenta | Early | Mid-resistant | 1996 |
| Iride | Southern Italy | Syngenta | Early | Mid-resistant | 1996 |
| Cysco | Northern Italy | Syngenta | Late | Resistant | 2001 |

**Table S2.** Compound discoverer settings for the metabolites search and confirmation.

| **Compound Discoverer settings** | |
| --- | --- |
| **Compound generator** |  |
| Parent Compound | T2 (C_24_H_34_O_9_) or HT2 (C_22_H_32_O_8_) |
| Phase I transformation | dehydration, desaturation, hydration, oxidation, reduction |
| Phase II transformation | acetylation, glucoside conjugation, methylation, cysteine conjugation, arginine conjugation, glycine conjugation, glutamine conjugation, GSH conjugation, sulfation |
| Maximum number of reactions | 3 |
| Ionization | [M+H]^+^ , [M+NH_4_]^+^ |
| **Expected finder** |  |
| Mass tolerance | 5 ppm |
| Intensity tolerance (isotope search) | 30% |
| Minimum peak intensity | 100000 |


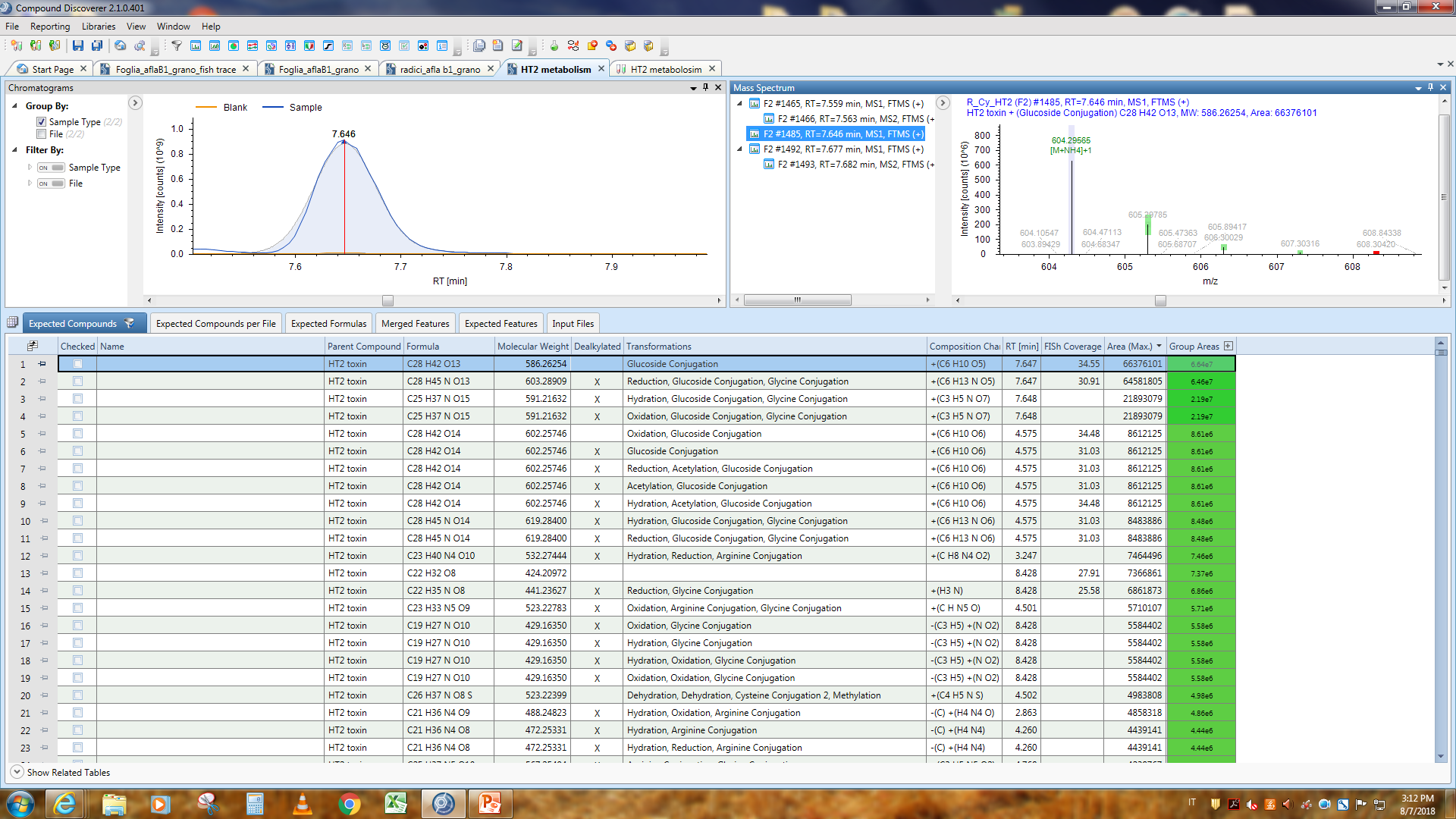


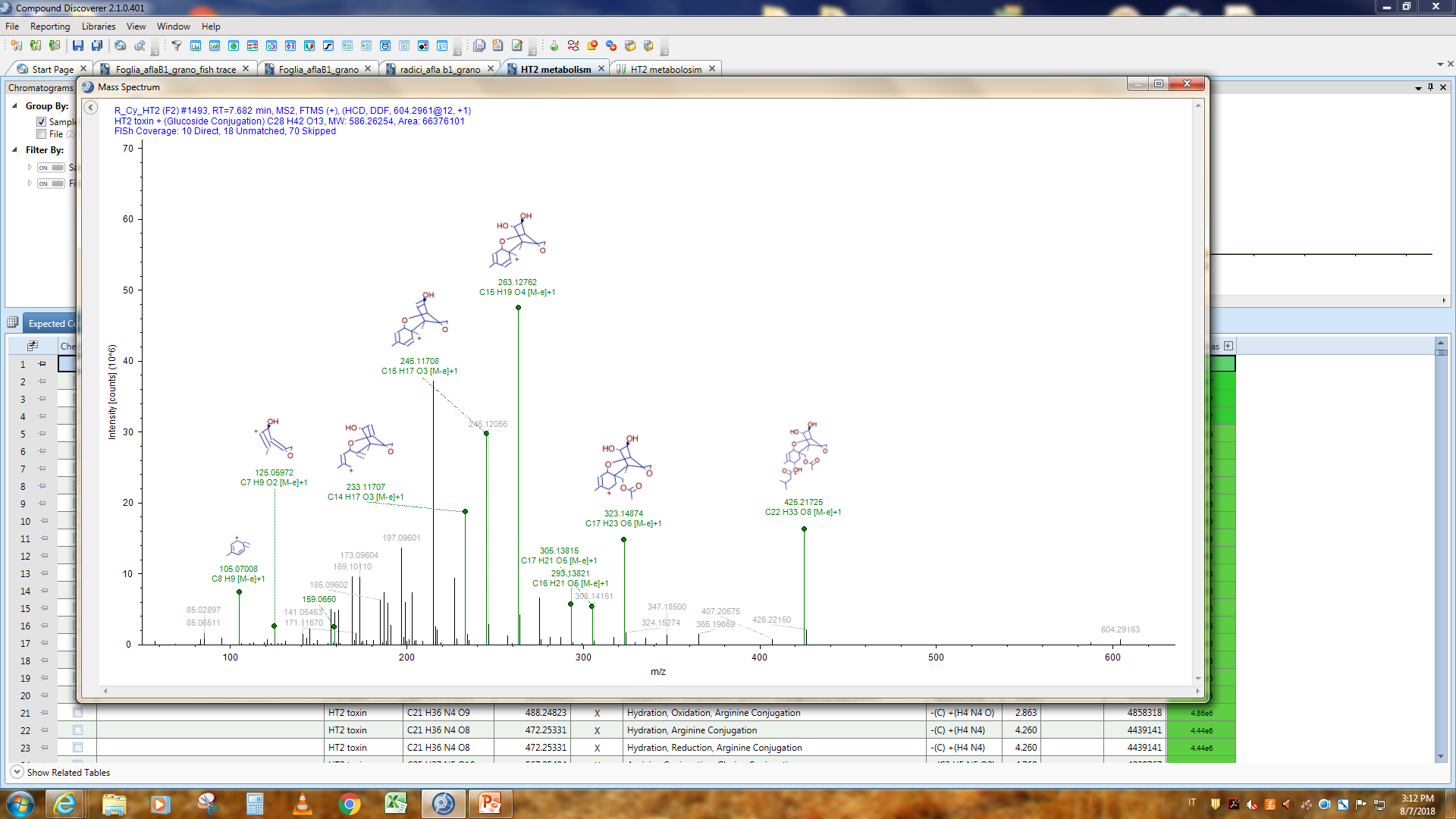


**Figure S3.** Example of identification steps used when working with Compound discoverer software. Identification of HT2-Glc in leaves treated with HT2. The picture shows the XIC chromatogram, MS isotopic pattern and HRMS/MS mass spectra.

**Table S3.** List of suspected metabolites that were also examined both in positive and negative ionization modes but that could not be confirmed.

| **Metabolites** | **Formula** |
| --- | --- |
| T2-Sulf | C_24_H_34_O_12_S |
| T2-GSH | C_34_H_51_N_3_O_15_S |
| T2-Cys-Glutammate | C_32_H_48_N_2_O_14_S |
| T2-Cys-Gly | C_29_H_44_N_2_O_12_S |
| T2-Cys | C_27_H_41_NO_11_S |
| coumaroyl-T2 | C_33_H_40_O_11_ |
| sinapoyl-T2 | C_35_H_44_O_13_ |
| vanillyl-T2 | C_32_H_40_O_12_ |
| syringyl-T2 | C_33_H_42_O_13_ |
| HT2-Sulf | C_22_H_32_O_11_S |
| HT2-GSH | C_32_H_49_N_3_O_14_S |
| HT2-Cys-Glutammate | C_30_H_46_N_2_O_13_S |
| HT2-Cys-Gly | C_27_H_42_N_2_O_11_S |
| HT2-Cys | C_25_H_39_NO_10_S |
